# Development and Cross-Validation of Anthropometric Predictive Equations to Estimate Total Body Fat Percentage in Adult Women in Sri Lanka

**DOI:** 10.1155/2020/2087346

**Published:** 2020-07-15

**Authors:** Nirmala Rathnayake, Gayani Alwis, Janaka Lenora, Sarath Lekamwasam

**Affiliations:** ^1^Department of Nursing, Faculty of Allied Health Sciences, University of Ruhuna, Galle, Sri Lanka; ^2^Department of Anatomy, Faculty of Medicine, University of Ruhuna, Galle, Sri Lanka; ^3^Department of Physiology, Faculty of Medicine, University of Ruhuna, Galle, Sri Lanka; ^4^Population Health Research Center, Department of Medicine, Faculty of Medicine, University of Ruhuna, Galle, Sri Lanka

## Abstract

Attempts have been made to estimate body fat using anthropometry, and most of them are country-specific. This study was designed to develop and cross-validate anthropometric predictive equations to estimate the total body fat percentage (TBFP) of Sri Lankan adult women. A cross-sectional study was conducted in Galle, Sri Lanka, with two groups: Group A (group for equation development) and Group B (cross-validation group) (*n* = 175 each) of randomly selected healthy adult women aged 30–60 years. TBFP (%) was quantified with total body DXA (TBFP_DXA_). Height (m), weight (kg), and skinfold thickness (SFT, mm) at six sites and circumferences (cm) at five sites were measured. In the first step, four anthropometric equations were developed based on the data obtained from multiple regression analyses (TBFP_DXA_ = dependent variable and anthropometric measurements and age = independent variables) with Group A. They were developed on the basis of circumferences (TBFP1), SFTs (TBFP2), circumferences and SFTs (TBFP3), and highly significant circumferences and SFTs (*r* ≥ 0.6) (TBFP4). In the second step, the newly developed equations were cross-validated using Group B. Three equations (TBFP1, TBFP2, and TBFP4) showed the agreement with cross-validation criteria. There were no differences between TBFP_DXA_ and TBFP estimated by these equations (*p* > 0.05). They showed higher measurement concordance with TBFP_DXA_; correlation between measured TBFP with DXA and estimated with TBFP1, TBFP2, and TBFP4, respectively, was 0.80 (*R*^2^ = 0.65, SEE = 3.10), 0.83 (*R*^2^ = 0.69, SEE = 2.93), and 0.84 (*R*^2^ = 0.72, SEE = 2.78). Three anthropometric measurements based on predictive equations were developed and cross-validated to satisfactorily estimate the TBFP in adult women.

## 1. Introduction

Obesity and overweight are serious health concerns associated with multiple disease conditions among women, both globally and locally [1, 2]. The prevalence of obesity is determined using anthropometry which is considered as surrogates of body fat mass, while they predict the subcutaneous fat mass or even the lean mass. Anthropometry is defined as “the study of the human body in terms of the dimensions of bone, muscle, and adipose (fat) tissue” [3]. It includes measurements of weight, stature (standing height), recumbent length, skinfold thickness (SFT), circumferences (head, waist, limb, etc.), limb lengths, and breadths (shoulder, wrist, etc.) [3]. Of them, body mass index (BMI) and waist-to-hip ratio (WHR) which are derived from basic anthropometric measurements and waist circumference (WC) are the commonly used surrogates of obesity [4].

Even though these anthropometric measurements are used to assess global adiposity in clinical and research settings due to the simplicity and low cost [5], the real definition of obesity is based on the total body fat percentage (TBFP) [6]. However, its application is mostly limited to research settings due to the restricted availability of required technology [7].

Dual-energy X-ray absorptiometry (DXA) estimates TBFP with a high accuracy and is considered as the gold standard reference method for estimating body composition [7]. However, radiation exposure and relatively high cost have restricted its availability. Equations that estimate TBFP based on simple anthropometric measurements such as weight, height, SFTs, and circumferences may overcome this shortcoming since they are simple, inexpensive, and practical [8].

Durnin–Womersley [9] and Jackson–Pollock [10] are the formulae commonly used to evaluate body composition worldwide. These equations, however, need calculation of body density prior to TBFP calculation. Further, they have been shown to be invalid in the Sri Lankan context [8] since the proportions of body segments depend not only on weight, age, and gender but also on the ethnicity, genetics, and lifestyle.

Accurate assessment of body composition including body fat in South Asians is important since Asians have a higher fat mass for a given BMI compared to Caucasians [2]. Further, the rising prevalence of noncommunicable diseases (NCDs) in Asia is linked with the increasing prevalence of obesity and overweight among middle-aged women [2]. They have poor health-seeking behaviors, more sedentary lifestyle, and improper dietary habits making them prone to obesity and NCDs [2]. The attention of public health care, clinical care providers and research scientists is less for these middle-aged women, since much attention is given for maternal health and child health among the women health services. Since this vulnerability of middle-aged women is closely linked with obesity, simple tool to assess the adiposity status could screen the risk groups at their early stages to prevent the associated morbidity.

Using equations developed in populations elsewhere to predict the adiposity is inappropriate since the inherited characteristics of populations may vary [11]. It is essential that country/ethnic-specific equations to estimate fat mass should be developed based on local data. Although two equations have been developed for Sri Lankan women for this purpose, they have focused only the adolescent girls [12] and women with a narrow age range (30–45 years) [8]. Therefore, in this study, we developed and cross-validated simple anthropometric predictive equations to estimate the TBFP of adult middle-aged women including both pre- and postmenopausal status.

## 2. Materials and Methods

### 2.1. Study Design, Subjects, and Setting

This descriptive cross-sectional study included 350 community-dwelling women aged 30–60 years, selected from the field study area (Bope-Poddala Medical Officer of Health Area) of the Faculty of Medicine, University of Ruhuna, Galle, in Southern Sri Lanka using a multistage cluster sampling technique. This area is a semiurban area which has socioeconomic characteristics and disease prevalence similar to national figures [13]. The study was conducted during the period from June 2015 to January 2017 as a part of the study project titled “Effects of menopause on bodily structure, functions and physical health” [14] which was focused on middle-aged women in pre- and postmenopausal status.

Of the 18 public health midwife's areas in the study area, three areas (Godakanda East, Kapuhempala, and Kalegana) were selected randomly to recruit women for the development of anthropometry equations (Stage 01; Group A, *n* = 175) and two other areas (Hapugala and Kahaduwawaththa) were selected randomly to recruit women for the study to cross-validate the developed equations (Stage 02; Group B, *n* = 175). When selecting the women, the distribution of women between different menopausal status (premenopausal women (PrMW) and postmenopausal women (PMW)) and age groups (aged 30–40, 41–50, and 51–60 years) was considered to be approximately equal.

Women who were pregnant or lactating, suffering from NCDs, acute or chronic medical conditions, and polycystic ovarian syndrome were excluded. Also women on hormone replacement therapy or hormonal contraceptives were excluded from the study.

### 2.2. Measured Variables

Sociodemographic details, age, and menopausal status were recorded. Menopausal status was considered on the self-stated menstrual history based on the classification of Stages of Reproductive Aging Workshop (STRAW) [15]. Body weight (kg) was measured to the nearest 0.1 kg using a digital weighing indicator (stadiometer) (NAGATA SCALE CO., LTD, Tainan, Taiwan) while wearing light clothes after the urinary bladder is empty. The standing height (m) was measured without footwear and recorded to the nearest 0.1 cm with the same stadiometer. BMI (kg/m^2^) was calculated as weight divided by square height. Limb circumferences (cm) at midupper arm (MUAC), midthigh (ThC), medial calf (CaC) on the right side of the body, and waist (WC) and hip (HC) circumferences were measured using a nonstretchable plastic measuring tape to the nearest 1 mm (Table 1). Each circumference was obtained in triplicate with the measurement consistency of 1 mm in each measurement. SFTs (mm) were measured over the triceps (TrSFT), biceps (BSFT), calf (CaSFT), thigh (ThSFT), suprailiac (SISFT), and subscapular (SCSFT) regions using Holtain skinfold caliper (Holtain Ltd, UK) to the nearest 0.2 mm on the right side of the body (Table 1). Each SFT was obtained in triplicate with the measurement consistency of 0.2 mm in each measurement. If the measurement consistency exceeds the expected values (1 mm for circumferences and 0.2 mm for SFT), another separate measurement was taken. The three measurements that were within the acceptable range were then averaged [16]. All the measurements were made by the same investigator to minimize the measurement errors, adhering to the standard protocols [17]. The precision errors (CV%) of AIs included in this study were determined by measuring 30 women twice in the same setting on the same day. Precision errors of anthropometric measurements (SFTs and circumferences) considered for this study ranged from 1.30% to 5.66% (TrSFT: ≤1.99%; BSFT: <1.82; SISFT: <2.10%; SCSFT: <2.21%; ThSFT: ≤3.04%; CaSFT: ≤5.66%; MUAC: ≤1.59%; ThC: ≤1.30% and CaC: ≤2.32%; WC: <≤0.32%; and HC: ≤0.31%).

DXA was used as the reference standard to quantify the TBFP. TBFP (total fat mass divided by total body mass, multiplied by 100) was measured with DXA scanner (Hologic Discovery W, Hologic Inc, Bedford, MA, USA) adhering to the manufacturer's guidelines. All scans were performed by the same technician who calibrated the device each scanning day. Analytical software (APEX™ analysis software) provided by the DXA manufacturer was used to analyze the TBFP.

### 2.3. Statistical Analyses

Data were analysed using SPSS version 20.0. Descriptive statistics, means (SD) or frequency (%), were used to describe data. Group comparison of continuous data was performed with the independent sample *t*-test and the group comparison of categorical data was performed with the chi-square test to evaluate the suitability for cross-validation. *p* value <0.05 was considered statistically significant.

Development of new equations: data from the 175 women in Group A were used for the development of new equations. Pearson correlation coefficients (*r*) were estimated between TBFP_DXA_ and anthropometric measurements to identify significant associations. The development of equations was based on few criteria:

  Step 1: all the correlated circumferences with TBFP
  Step 2: all the correlated SFTs with TBFP
  Step 3: all the correlated circumference and SFTs with TBFP
  Step 4: highly significant circumferences and SFTs with TBFP (*r* ≥ 0.60)

The variables were entered into multiple regression models in a “stepwise” manner in these four steps to remove the weakly associated variables with TBFP_DXA_. The collinearity between variables was verified by the variance inflation factor (VIF) and tolerance (*T*) values. Thus, VIF values <10 and tolerance values above 0.1 were considered as acceptable [18]. Age, weight, and height were entered as common variables to all steps. Based on the selected variables by the regression analysis, mathematical equations were developed.

Cross-validation of newly developed equations: for the cross-validation of the newly developed equations, 175 study participants assigned to Group B were used. Scatter plots and correlations between the TBFP predicted by the newly developed equations and TBFP_DXA_ were determined. Mean differences between TBFP measured and estimated with developed equations were compared by the paired sample *t*-test. If the TBFP measured and estimated with developed equations was not statistically different, additionally, determination coefficient (*R*^2^) and standard error of estimate (SEE) were determined with linear regression analysis for those equations. To consider the equations to be valid, the validation criteria described by Lohman [19] were used, i.e., the equations tested should not be significantly different from the reference standard (TBFP_DXA_), SEE should be low (<3.5), and *R*^2^ should be high (>0.6). The equations which satisfied the above criteria were further tested for repeatability with Bland–Altman plots, and limits of agreements were calculated (mean difference ± 1.96SD) [20].

In order to assess the adequacy of the sample, power of the study was estimated by post hoc compute achieved power analysis using the G^∗^ Power software version 3.1.9.2 [21].

### 2.4. Ethical Clearance

Ethics Review Committee, Faculty of Medicine, University of Ruhuna, Sri Lanka, granted ethical clearance for the study (Reference number: 24.09.2014:3.2). Each participant signed a written informed consent before providing the information, measuring AIs and body composition.

## 3. Results

Sociodemographic and basic characteristics of measured variables of Groups A and B are shown in Tables 2 and 3, respectively. The sociodemographic characteristics, age, menopausal status, anthropometric measurements, and TBFP of the two groups were not different (*p* > 0.05), indicating that two groups were similar with regard to their basic characteristics.

In Group A, age (*r*; 0.23, *p*=0.02) and all anthropometric measurements were studied; weight (*r*; 0.63, *p* < 0.001), height (*r*; 0.15, *p* < 0.001), WC (*r*; 0.64, *p* < 0.001), HC (*r*; 0.75; *r* < 0.001), limb SFT (*r* range; 0.45 to 0.63, *p* < 0.001), central SFT (*r*; 0.63, *p* < 0.001), and limb circumferences (*r*; 0.56 to 0.64, *p* < 0.001) showed positive correlations with TBFP_DXA_. HC (*r*; 0.75) among circumferences and TrSFT among SFTs (*r*; 0.64) (*p* < 0.001) showed the highest correlations with TBFP.

The equations developed to estimate the TBFP are given in Table 4. No significant or strong collinearity or multicollinearity was observed among independent variables (VIF < 10 and tolerance values above 0.1) (data not shown).

**Figure 1:**
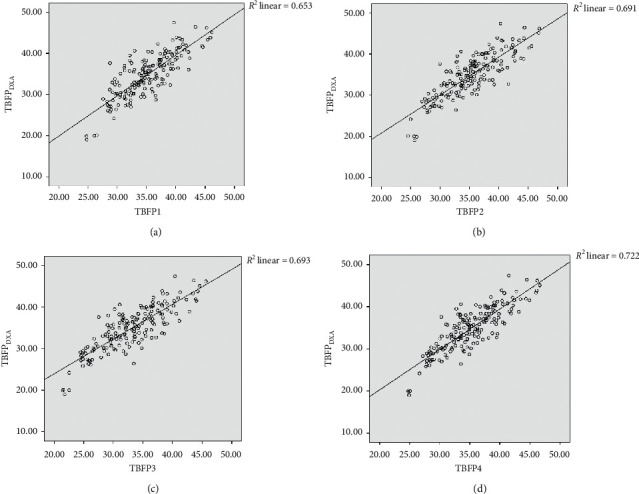
Correlation plotting between estimated TBFP by validated anthropometric equations and TBFP measured with DXA (TBFP_DXA_) (*n* = 175). (a) TBFP1 vs. TBFP_DXA_; (b) TBFP2 vs. TBFP_DXA_; (c) TBFP3 vs. TBFP_DXA_; (d) TBFP4 vs. TBFP_DXA_. TBFP = total body fat percentage.

**Figure 2:**
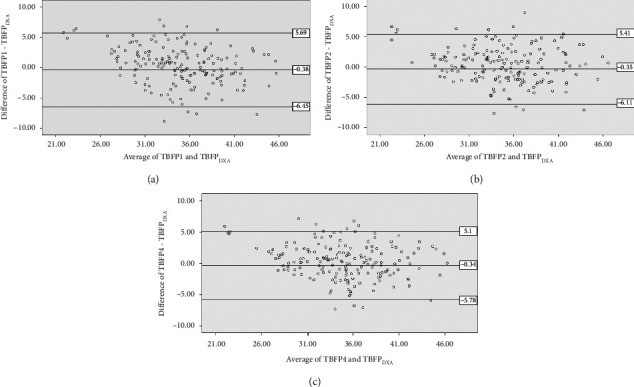
Agreement between newly developed equations and criterion method (TBFP_DXA_) (*n* = 175). (a) TBFP1; (b) TBFP2; (c) TBFP4. TBFP = total body fat percentage.

The results of the cross-validation are shown in Tables 5 and 6. Three equations (TBFP1, TBFP2, and TBFP4) met all the validation criteria; i.e., measured and estimated TBFPs were not significantly different (*p* < 0.05) (Table 5), and measured and estimated TBFPs had high correlations (*r* ranging from 0.80 to 0.84) and high coefficients of determination (65% to 72% variation of TBFP_DXA_) with low SEEs (2.78 to 3.10 kg) (Table 6). The equation derived in regression steps based on both circumferences and SFTs in combination with age, weight, and height (TBFP3) did not meet the validation criteria we followed (Table 5).  Figure 1 illustrates the scatter plots of measured and estimated TBFPs for the four equations. An acceptable measurement agreement (limits of agreements are shown in Table 5) was observed for equations TBFP1, TBFP2, and TBFP4 when data were examined by the Bland–Altman plots (Figure 2).

In the post hoc power calculation test, to calculate the sample power of valid anthropometry equations was conducted by adopting an error probability of 5% for the sample size used. The sample power (1-*β* error probability) was 1.00 for all the valid equations.

## 4. Discussion

Of the four anthropometric predictive equations developed, three met all the validation criteria we followed; measured and estimated TBFPs were not significantly different and measured and estimated TBFPs had high correlations and high coefficients of determination with low SEEs. The validity of these equations to predict TBFP in adult women is indicated by the high concordance between the TBFP measured with DXA and calculated with equations with acceptable measurement agreement and only few variables were beyond the limits of agreements. Of the equations we developed, the equation based on body circumferences may be a more feasible approach than that based on SFTs since measurement of SFT needs special equipment and training compared to the measuring of circumferences. However, compared to others, the equation we developed incorporating both circumferences and SFTs which had higher correlations with TBFP showed a greater variance of TBFP that would be due to the higher representation of body sites in the equation.

Concordant with the current study findings, SFTs such as TrSFT, CaSFT, and SISFT [22–24] and circumferences such as WC and HC [25–27] have shown significant correlations with TBFP in studies done elsewhere. The equations developed based on the circumferences or SFTs in combination with weight, height, and age have shown the greater variance of TBFP [22–27] in adult women. Even though the higher variances (>50%) between the measured and predictive TBFPs were observed among the studies, significant deviations were observed. This could be attributed to the differences in the distribution of body composition and lifestyle patterns of different groups of women, sample size, and the method used to measure the TBFP such as DXA [27], bioelectrical impedance analysis [25], hydrodensitometry [22], or compartment model [24].

A Sri Lankan study [12] has shown that SFTs predicted TBFP better in postmenarcheal girls aged between 15 and 19 years and the developed predictive equation was based on TrSFT and SISFT. However, this equation has shown poor predictive abilities when applied to women aged 30–45 years [8]. This might be due to that postmenarcheal girls have less body fat compared to the women in reproductive ages and beyond which limits the applicability of equations to women in a wider age range. Therefore, an attempt has been made to develop an equation for middle-aged women, and this equation includes only the TrSFT and weight [8]. The equation has developed with the women aged 30–45 years [8] and used weight, WC, BSFT, TrSFT, SCSFT, and SISFT as independent variables while only TrSFT and weight have been incorporated into the equation based on the regression analysis results. Circumferences have been disappeared with the regression analysis even though they are direct measures of central obesity. However, a greater variance of TBFP has been observed with the predictive equation; the equation has been developed and cross-validated using a group of women with a narrow age range (30–45 years). Apart from that, H_2_O dilution with Fourier transform infrared spectroscopy has been used as the reference method for detecting TBFP in these two studies [8, 12]. Therefore, sample selection, absence of the circumferences in the developed equation, and the reference method used might lessen the wide applicability of developed equations previously in Sri Lanka. Compared to them, the importance of our equations is that they are applicable to adult women with a wider age range representing both pre- and postmenopausal women, easy to apply, derived against a gold standard reference method (DXA), and have shown higher accuracy since they represent both SFTs and circumferences. Further, we selected an equal number of women for two groups which represent different age groups and menopausal statuses since age and menopausal status are the two major biological confounder effects on body fat content and anthropometry [28]. Therefore, equations are fairly applicable to both aging and menopausal variations as well. Further, our study samples were selected randomly from a semiurban area in Sri Lanka which has socioeconomic characteristics and disease prevalence similar to national figures [13]. These qualities enhance the applicability of equations we developed to the entire adult women population in all parts of the country. Importantly, the applications of these equations totally depend on individual users, which will be decided based upon the available or easily measurable AIs with the available resources at the particular setting.

Our study has several strengths and limitations. We measured the TBFP with a gold standard technique, and measurement of anthropometry was done by a trained personal with low precision errors between measurements. Furthermore, random selection of women from the representative population of Sri Lanka, selection of similar groups for the development, and cross-validation of equation with the special concern on sociodemographic characteristics, age, and menopausal status are a few strengths of the current study. However, this study included only adult middle-aged women, and our findings cannot be applied to women in other extremes of ages and men, where the further studies are required.

## 5. Conclusions

In this study, we developed and validated simple predictive equations using different anthropometric measurements to quantify the TBFP of adult Sri Lankan women. Out of the entire equations developed, the one which includes SFTs and circumferences (TBFP4) has the highest measurement concordance. However, three equations that met all validation criteria have high accuracy and proved that they are suitable for research and clinical settings. The selection of the equation can be based on the availability of resources for measuring the anthropometry in the particular setting.

## Figures and Tables

**Table 1:** Measurement landmarks of circumferences and SFTs [3, 16].

Site of circumference or SFT	Landmarks of measurements
MUAC	Measured at midway between the lateral projection of the acromion process of the scapula and the inferior margin of the olecranon process of the ulna
ThC	Measured at midway between the midpoint of the inguinal crease and the proximal border of the patella
CaC	Measured at the maximal circumference of the calf
WC	Measured at midway between iliac crest and lower rib margin at the end of normal expiration
HC	Measured at the widest part of the buttocks at intertrochantric level
TrSFT	Measured at the vertical fold of posterior midline of the upper arm, halfway between the acromion (shoulder) and olecranon processes (elbow), while the arm held freely at the side of the body
BSFT	Measured at the vertical fold, anterior aspect of the arm over the belly of the biceps muscle, 1 cm above the level used to mark the triceps site
ThSFT	Measured at the vertical fold anterior midline of the thigh, midway between the proximal border of the patella (upper knee) and the inguinal crease (hip)
CaSFT	Measured at the vertical fold, maximum circumference of the calf on the midline of the medial border.
SISFT	Measured at the diagonal fold, anterior axillary line; immediately superior to the iliac crest, in line with the natural angle of the iliac crest taken, midaxillary line (traditional technique), superior to the iliac crest
SCSFT	Measured at the diagonal fold, 1 to 2 cm below the inferior angle of the scapula

MUAC = midupper arm circumference; CaC = calf circumference; ThC = thigh circumference; WC = waist circumference; HC = hip circumference; TrSFT = triceps skinfold thickness; BSFT = biceps skinfold thickness; CaSFT = calf skinfold thickness; ThSFT = thigh skinfold thickness; SISFT = suprailiac skinfold thickness; SCSFT = subscapular skinfold thickness.

**Table 2:** Sociodemographic characteristics of women in Groups A and B (*n* = 350).

Characteristics	Subcategory	Group A (*n* = 175) frequency (%)	Group B (*n* = 175) frequency (%)
Age	Aged 30–40 years	40 (22.9)	40 (22.9)
	Aged 41–50 years	50 (28.6)	56 (32.0)
	Aged 51–60 years	85 (48.6)	79 (45.1)

Menopausal status	PrMW	92 (52.6)	92 (52.6)
	PMW	83 (47.4)	83 (47.4)

Ethnicity	Sinhala	166 (94.9)	165 (94.3)
	Non-Sinhala	9 (5.1)	10 (5.7)

Employment status	Employed	56 (32.0)	51 (29.1)
	Unemployed	119 (68.0)	124 (70.9)

Civil status	Married	145 (82.9)	148 (84.6)
	Single or widowed or divorced	30 (17.1)	27 (15.4)

Living companion	With husband and children	119 (68.0)	116 (66.3)
	With husband or children	29 (16.6)	23 (66.3)
	Alone or living with others	27 (15.4)	36 (20.6)

Education status	Primary education	45 (27.5)	38 (21.7)
	Secondary education	62 (35.4)	70 (40.0)
	Upper secondary or tertiary education	68 (38.9)	67 (38.3)

Monthly income	Below 20000 LKR	106 (60.6)	110 (62.9)
	Above 20000 LKR	69 (39.4)	65 (37.1)

LKR = Sri Lankan rupees (190LKR = 1USD); PrMW = premenopausal women; PMW = postmenopausal women. Living with others includes parents, siblings, friends, or other relatives.

**Table 3:** Basic characteristics of women in Groups A and B (*n* = 350).

Characteristic	Group A (*n* = 175) mean (SD)	Group B (*n* = 175) mean (SD)
Age (years)	48.7 (8.5)	48.7 (8.3)
Weight (kg)	58.10 (10.93)	57.04 (10.84)
Height (m)	1.51 (0.60)	1.50 (0.06)
WC (cm)	83.22 (10.30)	83.08 (9.95)
HC (cm)	97.91 (9.45)	97.83 (9.34)
MUAC (cm)	30.89 (3.86)	30.91 (3.73)
CaC (cm)	33.35 (3.33)	33.05 (3.39)
ThC (cm)	49.82 (6.09)	49.98 (6.25)
TrSFT (mm)	19.21 (6.18)	19.78 (6.23)
BSFT (mm)	9.86 (4.50)	10.53 (5.00)
CaSFT (mm)	17.80 (7.07)	17.98 (8.06)
ThSFT (mm)	27.32 (9.66)	27.51 (9.59)
SISFT (mm)	16.02 (10.66)	16.41 (10.52)
SCSFT (mm)	20.03 (7.26)	20.43 (7.31)
BMI (kg/m^2^)	25.23 (4.24)	25.12 (4.34)
WHR	0.84 (0.05)	0.84 (0.05)
TBFP (%)	34.88 (5.35)	34.79 (5.26)

SD = standard deviation; WC = waist circumference; HC = hip circumference; MUAC = midupper arm circumference; CaC = calf circumference; ThC = thigh circumference; TrSFT = triceps skinfold thickness; BSFT = biceps skinfold thickness; CaSFT = calf skinfold thickness; ThSFT = thigh skinfold thickness; SISFT = suprailiac skinfold thickness; SCSFT = subscapular skinfold thickness; BMI = body mass index; WHR = waist-to-hip ratio; TBFP = total body fat percentage; PrMW = premenopausal women; PMW = postmenopausal women.

**Table 4:** Equations developed to estimate TBFP of middle-aged women in Group A (*n* = 175).

Step	Basis of equation	Equation		*r* ^*∗*^	*R* ^2^	SEE
1	Circumferences	TBFP1	32.484 + 0.464 (HC) − 28.385 (height)	0.81	0.66	3.12
2	SFTs	TBFP2	44.623 + 0.154 (age) + 0.207 (weight) − 24.118 (height) + 0.163 (ThSFT) + 0.092 (SISFT) + 0.141 (BSFT)	0.85	0.71	2.84
3	Circumferences and SFTs	TBFP3	23.286 + 0.259 (HC) − 15.449 (height) + 0.172 (ThSFT) + 0.123 (age) + 0.074 (SISFT) − 0.27 (CaC) + 0.137 (BSFT) + 0.112 (ThC)	0.87	0.76	2.65
4	Highly significant circumferences and SFTs	TBFP4	23.657 + 0.297 (HC) − 19.261 (height) + 0.175 (ThSFT) + 0.113 (age) + 0.065 (SISFT)	0.86	0.73	2.76

TBFP = total body fat percentage; SFT = skinfold thickness; HC = hip circumference; CaC = calf circumference; ThC = thigh circumference; BSFT = biceps skinfold thickness; ThSFT = thigh skinfold thickness; SISFT = suprailiac skinfold thickness. *r* = correlation coefficient, *R*^2^ = determination coefficient, and SEE = standard error of estimate. Weight in kg, height in m, circumference in cm, and SFT in mm. ^*∗*^Correlations were significant at <0.001 level.

**Table 5:** Cross-validation of developed equations; comparison of measured and estimated TBFP in Group B (*n* = 175).

Model	Mean (SD) (kg)	Mean difference (SD)	Range of mean difference	Standard error mean	Significance (*p* value)
TBFP1	35.13 (4.31)	−0.38 (3.10)	−0.85 to 0.07	0.23	0.10
TBFP2	35.10 (4.73)	−0.35 (2.94)	−0.79 to 0.08	0.22	0.11
TBFP3	32.90 (5.22)	1.84 (3.03)	1.39 to 2.30	1.22	<0.001
TBFP4	35.09 (4.66)	−0.34 (2.78)	−0.76 to 0.06	0.21	0.09

TBFP = total body fat percentage; SD = standard deviation. Mean comparison was performed with the paired sample *t*-test.

**Table 6:** Cross-validation of developed equations; regression analysis with Group B and limits of agreements (*n* = 175).

Equation	Regression analysis			Limits of agreements (mean difference ± 1.96 SD)
	Correlation coefficient (*r*)^*∗*^	Determination coefficient (*R*^2^)	Standard error of estimate (SEE)	
TBFP1	0.80	0.65	3.10	−6.45 to 5.69
TBFP2	0.83	0.69	2.93	−6.11 to 5.41
TBFP4	0.84	0.72	2.78	−5.78 to 5.10

TBFP = total body fat percentage; SD = standard deviation. *r* = correlation coefficient, *R*^2^ = determination coefficient, and SEE = standard error of estimate. ^*∗*^Correlations are significant at <0.001.

## Data Availability

The data used to support the findings of this study are available from the corresponding author upon request.

## References

[1] Ogden C. L., Carroll M. D., Fryar C. D., Flegal K. M. (2015). *Prevalence of Obesity Among Adults and Youth: United States, 2011–2014*.

[2] Katulanda P., Jayawardena M. A. R., Sheriff M. H. R., Constantine G. R., Matthews D. R. (2010). Prevalence of overweight and obesity in Sri Lankan adults. *Obesity Reviews*.

[3] Centers for Disease Control and Prevention (2007). *National Health and Nutrition Examination Survey (Nhanes): Anthropometry Procedures Manual*.

[4] WHO Expert Committee (2004). Appropriate body-mass index for Asian populations and its implications for policy and intervention strategies. *The Lancet*.

[5] Wang J., Thornton J., Kolesnik S., Pierson R. (2000). Anthropometry in body composition: an overview. *Annals of the New York Academy of Sciences*.

[6] Gallagher D., Heymsfield S. B., Heo M., Jebb S. A., Murgatroyd P. R., Sakamoto Y. (2000). Healthy percentage body fat ranges: an approach for developing guidelines based on body mass index. *The American Journal of Clinical Nutrition*.

[7] Lee S. Y., Gallagher D. (2008). Assessment methods in human body composition. *Current Opinion in Clinical Nutrition and Metabolic Care*.

[8] Waidyatilaka I., de Silva A., de Lanerolle-Dias M., Atukorala S., Lanerolle P. (2016). A field tool for prediction of body fat in Sri Lankan women: skinfold thickness equation. *Journal of Health, Population and Nutrition*.

[9] Durnin J. V. G. A., Rahaman M. M. (1967). The assessment of the amount of fat in the human body from measurements of skinfold thickness. *British Journal of Nutrition*.

[10] Jackson A., Stanforth P. R., Gagnon J. (2002). The effect of sex, age and race on estimating percentage body fat from body mass index: the heritage family study. *International Journal of Obesity*.

[11] Leahy S., O’Neill C., Sohun R., Toomey C., Jakeman P. (2013). Generalised equations for the prediction of percentage body fat by anthropometry in adult men and women aged 18–81 years. *British Journal of Nutrition*.

[12] de Lanerolle-Dias M., de Silva A., Lanerolle P., Arambepola C., Atukorala S. (2011). Body fat assessment in Sri Lankan adolescent girls; development of a simple field tool. *Annals of Human Biology*.

[13] Wijewardene K., Mohideen M. R., Mendis S. (2005). Prevalence of hypertension, diabetes and obesity: baseline findings of a population based survey in four provinces in Sri Lanka. *The Ceylon Medical Journal*.

[14] Rathnayake N., Lenora J., Alwis G., Lekamwasam S. (2019). Prevalence and severity of menopausal symptoms and the quality of life in middle-aged women: a study from Sri Lanka. *Nursing Research and Practice*.

[15] Harlow S. D., Gass M., Hall J. E. (2012). Executive summary of the stages of reproductive aging workshop + 10: addressing the unfinished agenda of staging reproductive aging. *The Journal of Clinical Endocrinology & Metabolism*.

[16] Eckel R. H. (2003). *Obesity: Mechanisms and Clinical Management*.

[17] Lohman T. G. (1988). Anthropometry and body composition. *Anthropometric Standardization Reference Manual*.

[18] Snee R. D. (1977). Validation of regression models: methods and examples. *Technometrics*.

[19] Lohman T. G. (1992). *Advances in Body Composition Assessment*.

[20] Bland J. M., Altman D. (1986). Statistical methods for assessing agreement between two methods of clinical measurement. *The Lancet*.

[21] Faul F., Erdfelder E., Lang A.-G., Buchner A. (2007). G^∗^ power 3: a flexible statistical power analysis program for the social, behavioral, and biomedical sciences. *Behavior Research Methods*.

[22] Aristizabal J. C., Estrada Restrepo A., Giraldo García A. (2018). Development and validation of anthropometric equations to estimate body composition in adult women. *Colombia Médica*.

[23] Ojo G., Adetola O. (2017). The relationship between skinfold thickness and body mass index in estimating body fat percentage on Bowen University students. *International Biological and Biomedical Journal*.

[24] Peterson M. J., Czerwinski S. A., Siervogel R. M. (2003). Development and validation of skinfold-thickness prediction equations with a 4-compartment model. *The American Journal of Clinical Nutrition*.

[25] Raimi T., Oluwayemi I. (2017). Anthropometric correlates and prediction of body fat measured by bioelectric impedance analysis among women. *Annals of Medical and Health Sciences Research*.

[26] Salamunes A. C. C., Stadnik A. M. W., Neves E. B. (2018). Estimation of female body fat percentage based on body circumferences. *Revista Brasileira de Medicina do Esporte*.

[27] Lahav Y., Epstein Y., Kedem R., Schermann H. (2018). A novel body circumferences-based estimation of percentage body fat. *British Journal of Nutrition*.

[28] Trémollieres F. A., Pouilles J.-M., Ribot C. A. (1996). Relative influence of age and menopause on total and regional body composition changes in postmenopausal women. *American Journal of Obstetrics and Gynecology*.

